# Managing Laryngeal Carcinoma in the United Arab Emirates: An Experience From the Tertiary Care Center

**DOI:** 10.7759/cureus.89563

**Published:** 2025-08-07

**Authors:** Reem Aldhaheri, Shayan Ansari, Rana Alkatheeri, Mariam Alshamsi, Latifa Alhajeri, Ameera Alghafri

**Affiliations:** 1 Otolaryngology, Tawam Hospital, Al Ain, ARE

**Keywords:** chemoradiotherapy, laryngeal cancer, retrospective study, surgical treatment, survival analysis, treatment outcomes

## Abstract

Background

Laryngeal cancer is a common head and neck malignancy. The treatment modalities differ depending on the clinical staging and location of the tumor, hence affecting the survival outcomes.

Objective

This study aims to provide demographic information and survival outcomes in laryngeal cancer patients in the UAE, considering the clinical staging and treatment received.

Methods

A retrospective cohort study in laryngeal cancer patients at Tawam Hospital from 2016 to 2020. Descriptive analysis summarized the demographics and survival analysis using Kaplan-Meier curves, comparing outcomes between early and advanced stages, as well as treatment modality (surgical vs non-surgical). We also used the log-rank test to assess statistical significance.

Results

A total of 63 patients were included in the study. Patients with early-stage disease showed better survival in the surgical compared to the non-surgical primary treatment. On the contrary, patients with advanced-stage disease had better survival outcomes in the non-surgical treatment group than in the surgical group.

Conclusion

Compared to early-stage disease, the treatment modality does affect the survival outcomes in advanced stages. These results suggest a potential benefit favoring the non-surgical treatment modality, but further prospective studies with larger sample sizes are needed.

## Introduction

Laryngeal cancer accounts for significant morbidity and mortality worldwide. In 2020, the global incidence of laryngeal cancer was estimated at 184,615 new cases, with approximately 99,840 deaths attributed to the disease that year [1].

It is classified based on the laryngeal subunit involved. This includes the supraglottic, glottic, and subglottic regions, with the glottic region accounting for the majority of cases, followed by the supraglottic and subglottic regions. Squamous cell carcinoma is the most common histopathological diagnosis. Tobacco smoking and alcohol intake are two of the most well-established risk factors associated with laryngeal cancer [2-4].

The treatment for laryngeal cancer depends on the stage and location of the tumor. Early-stage disease may be managed with single-modality treatment such as primary surgery, radiotherapy, or chemotherapy. In contrast, advanced-stage disease often requires multimodal treatment, which may include a combination of surgery, radiation therapy, and/or chemotherapy. There is ongoing debate regarding the optimal treatment approach for advanced-stage disease in terms of survival outcomes [5-8].

Given the limited data and research available from the United Arab Emirates (UAE), the primary objective of this retrospective study was to assess overall survival in patients with laryngeal cancer treated at our center, comparing outcomes between those who received primary surgical treatment and those who received primary non-surgical treatment, including chemoradiotherapy, radiotherapy alone, chemotherapy alone, or palliative care. Secondary objectives included a descriptive analysis of patient demographics, an evaluation of the distribution of treatment modalities by disease stage, and an examination of survival trends across stage groups. Patients with unknown follow-up status or those who received palliative care or salvage surgery were excluded from the survival analysis to maintain consistency and accuracy in time-to-event data.

## Materials and methods

Our retrospective cohort study was conducted at Tawam Hospital, a tertiary care center in Al Ain, United Arab Emirates. Data were collected from the cancer registry at Tawam Hospital. The medical records of patients diagnosed with laryngeal cancer between January 2016 and December 2020 were manually reviewed to confirm the data.

The study population included all patients diagnosed with laryngeal cancer during the study period. Inclusion criteria included the histopathological confirmation of laryngeal cancer, documented clinical staging, and the available survival follow-up data.

Patients with incomplete records regarding staging or survival outcomes were excluded from the analysis. The variables that were collected included demographics: age at diagnosis, gender, smoking status (categorized as smoker, non-smoker, or unknown), and clinical characteristics, specifically the tumor subunit (supraglottic, glottic, subglottic, transglottic). Clinical staging was based on the American Joint Committee on Cancer (AJCC) 8th edition guidelines [1], and the patients were classified into early stage (Stages I-II) and advanced stage (Stages III-IV). The primary treatment the patient received included palliative radiotherapy alone, chemotherapy alone, combined chemoradiotherapy, and primary surgery. The outcome was noted as overall survival in months from diagnosis to death or last follow-up or status at last follow-up (alive or deceased).

Data analysis was conducted using IBM SPSS Statistics for Windows, Version 26.0 (IBM Corp., Armonk, NY, US). Descriptive statistics summarized demographic and clinical variables. Categorical variables were presented as counts and percentages; continuous variables were presented as means and standard deviations.

Kaplan-Meier survival analysis was performed to estimate overall survival, stratified by clinical stage, which was grouped as early (Stages I-II) and advanced (Stages III-IV), and treatment received (grouped as primary surgical and primary non-surgical). Patients were censored at the last known date of follow-up to check if they were still alive. In the survival analysis, patients whose recurrence status was labeled as unknown due to loss of follow-up, patients who were never disease-free, and patients who underwent palliative therapy were excluded. Other survival endpoints (e.g., disease-free survival, progression-free survival) were not included due to data limitations inherent to the retrospective nature of the study.

## Results

A total of 63 patients met the inclusion criteria. The median age was 61 years. The majority of cases were male, accounting for 61 (96.8%). Smoking status was known for 47 (74.6%) patients, of whom 41 (65.1%) were smokers. Among patients with known smoking status, only two were also documented as alcohol users. However, alcohol use was not consistently recorded across the cohort, and data were missing for the majority of cases.

**Figure 1:**
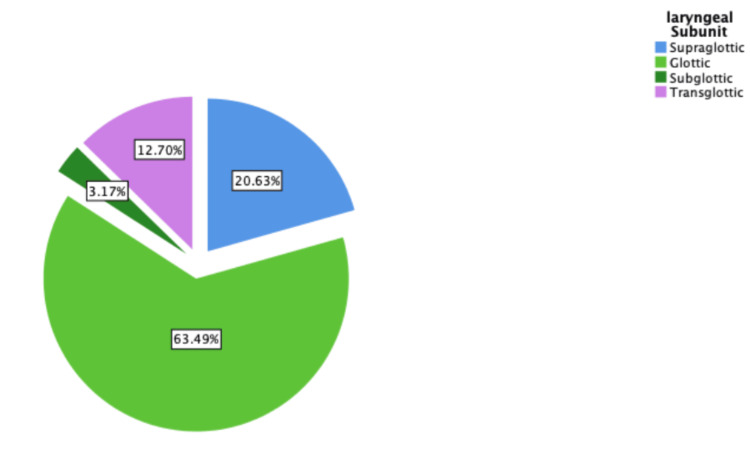
Laryngeal subunits involved, labeled with percentages The figure demonstrates higher involvement of the glottic subunit.

The most affected subunit of the larynx was the glottic region, accounting for 40 (63.5%) of all cases. The supraglottic region was involved in 13 (20.6%) patients, while subglottic involvement was seen in 2 (3.2%). Transglottic tumors were observed in 8 (12.7%) patients (Figure 1).

According to clinical tumor, node, metastasis (TNM) staging, 23 (36.5%) patients were classified as early stage: 14 (60.9%) of these were Stage I and 9 (39.1%) were Stage II. The remaining 40 (63.5%) patients were classified as advanced stage: 18 (45.0%) in Stage III and 22 (55.0%) in Stage IV.

**Figure 2:**
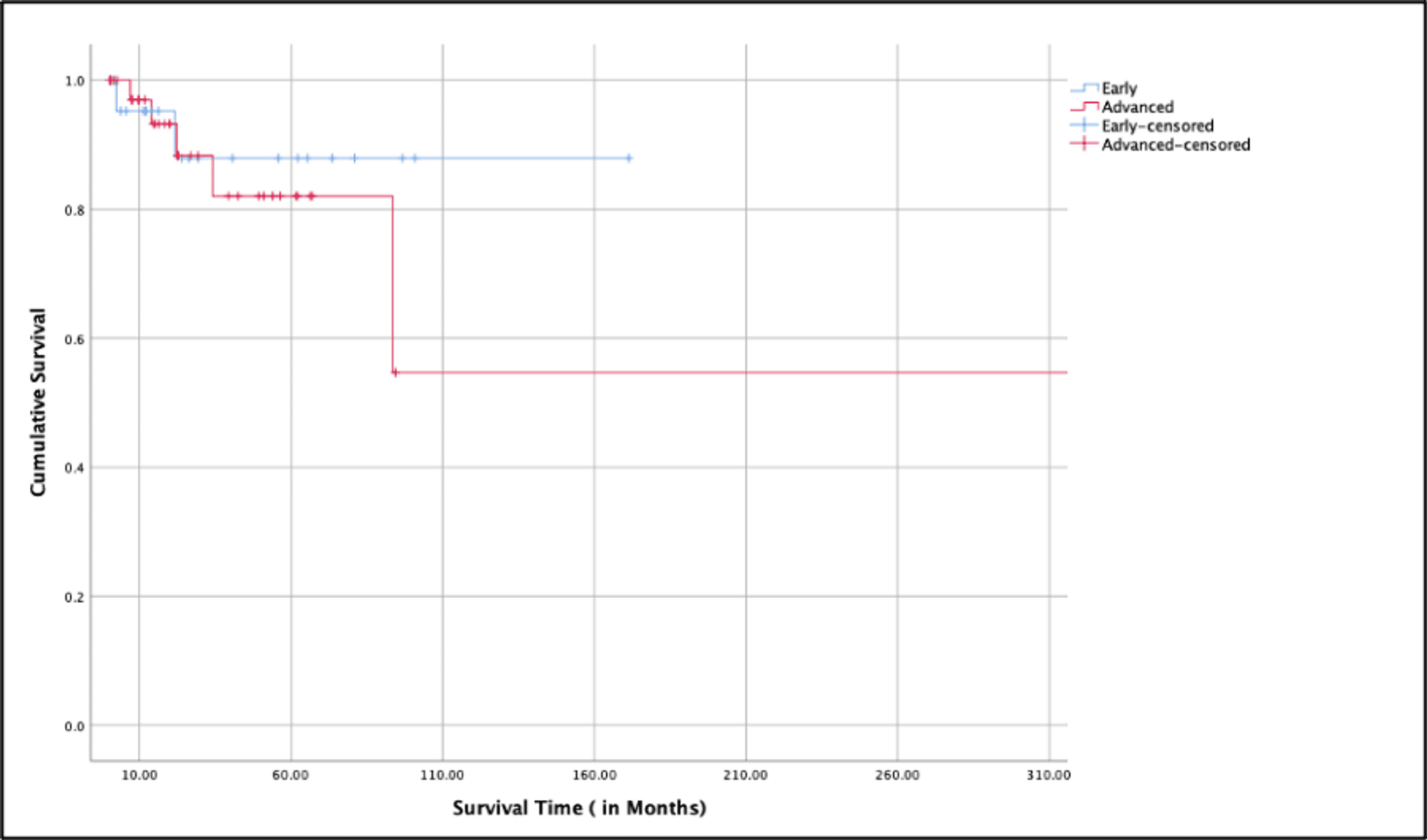
Kaplan-Meier survival analysis between early vs advanced laryngeal cancer cases

Using Kaplan-Meier survival analysis showed that patients with early-stage laryngeal cancer had a significantly longer median survival than those with advanced-stage disease. The cumulative survival curve showed higher survival rates in the early-stage group. Patients were censored at the last known date of follow-up if they were still alive (Figure 2).

The cohort was divided into five groups based on the primary treatment received: primary surgical treatment, radiotherapy, chemoradiotherapy, chemotherapy alone, and palliative therapy. A total of 14 (22.2%) patients received primary surgical treatment alone, 23 (36.5%) received chemoradiotherapy, 20 (31.7%) received radiotherapy, 2 (3.2%) received chemotherapy alone, and 4 (6.3%) received palliative treatment.

**Figure 3:**
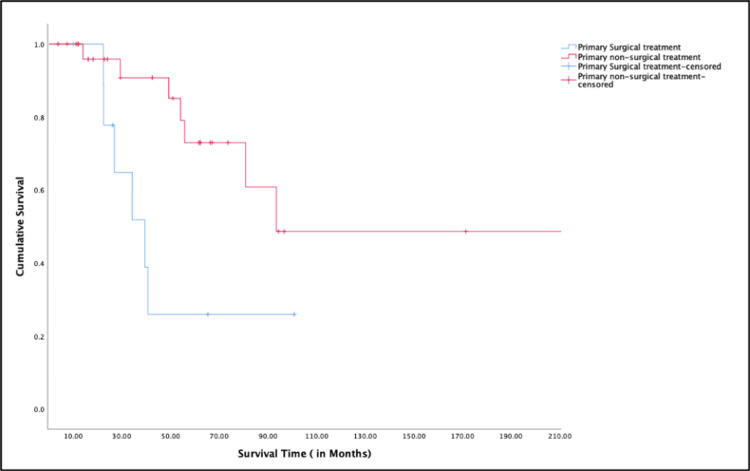
Kaplan-Meier survival analysis between patients between surgical vs. non-surgical treatment

The treatment was grouped into two main categories: primary surgical versus primary non-surgical. Kaplan-Meier survival analysis was also done comparing primary surgical treatment versus primary non-surgical treatment (Figure 3).

**Figure 4:**
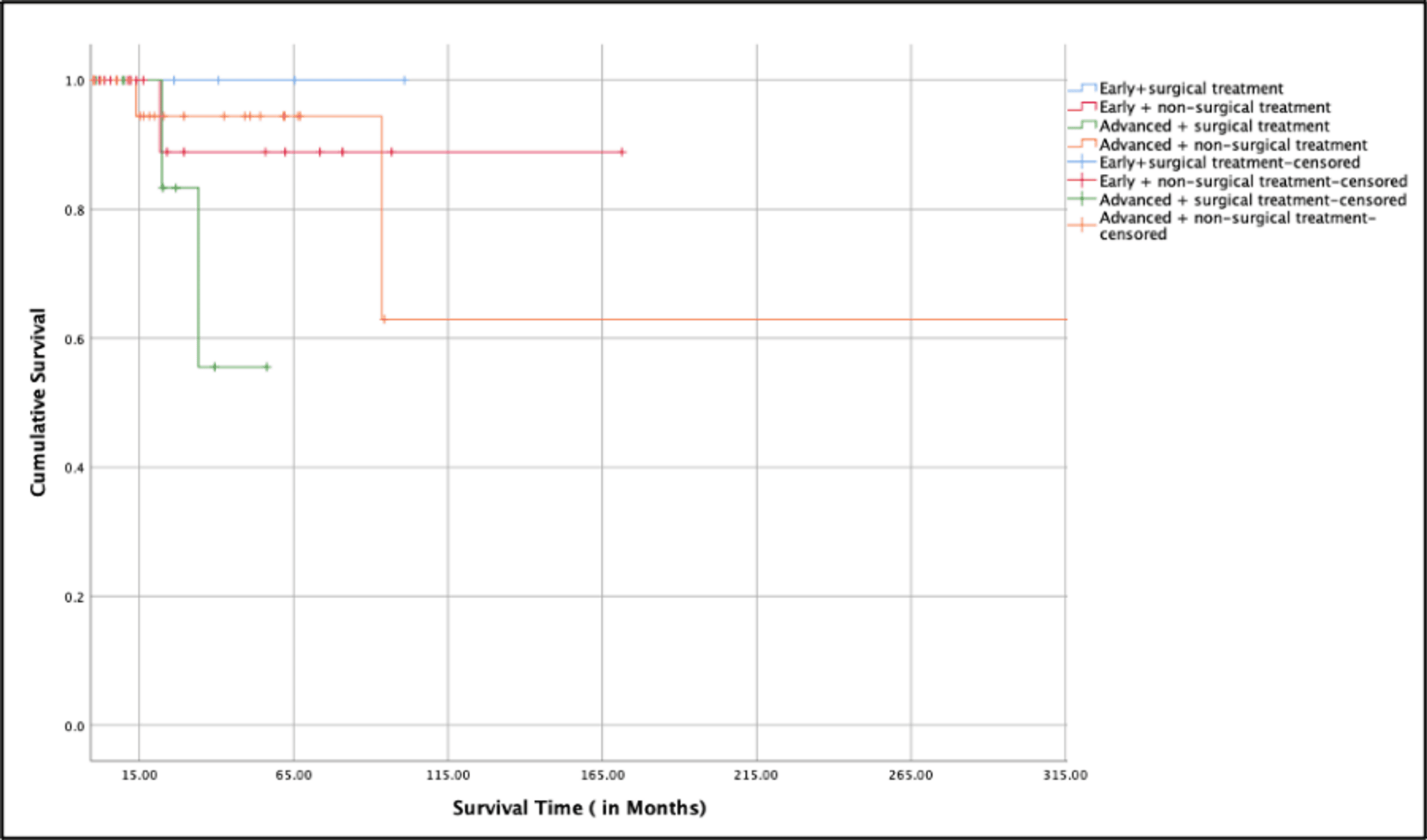
Both early-stage groups (surgical and nonsurgical) show excellent survival, while the advanced + surgical group shows the lowest survival

It was notable that patients who received primary non-surgical treatment had better cumulative survival rates over time compared to those who had primary surgical treatment. Kaplan-Meier survival analysis was also performed to evaluate survival across different combinations of clinical stage (early vs. advanced) and primary treatment modality (surgical vs. non-surgical). The analysis revealed that both early-stage groups (whether treated surgically or non-surgically) demonstrated excellent long-term survival, with cumulative survival rates remaining close to 100% over time. In contrast, patients in the advanced-stage surgical treatment group exhibited the lowest survival, showing a rapid decline in cumulative survival within the first few months. The advanced-stage non-surgical group showed intermediate survival outcomes (Figure 4).

Notably, of all patients with advanced disease who received primary non-surgical treatment, only four patients underwent salvage total laryngectomy. All patients with advanced-stage disease who had primary surgical treatment underwent post-operative chemoradiotherapy.

## Discussion

The demographic observed in this study correlates with the established worldwide demographic findings of laryngeal cancer [2,3]. This includes the age range between the late 40s and 60s. The predominant gender was male gender in 61 (96.8%) of patients. 

Smoking and alcohol are known risk factors for laryngeal cancer [4,5]. It was observed that smoking was known as a risk factor in 47 (74.6%) of patients, 6 (9.5%) were known to be non-smokers, and 16 (25.4%) patients' smoking status was unknown. Among patients with documented alcohol use, only 2 (3.1%) were identified as alcohol users. However, alcohol use was not consistently recorded across the cohort, and data were missing for the majority of cases. This reflects a limitation in data completeness and highlights the importance of standardized documentation in oncology cases.

Survival was noted to be better in the non-surgical treatment group and among those with early-stage disease. Other confounding factors may contribute to these findings, including patient co-morbidities, tumor stage, and the selection criteria for the treatment modality. These findings may indicate a potential trend favoring non-surgical treatment modalities in certain cases; however, given the retrospective design, small sample size, and lack of multivariable analysis, this observation should be interpreted as exploratory and hypothesis-generating rather than definitive.

In our study, about 40 (63.49%) of cases were in advanced-stage laryngeal cancer, and our results showed that there is clear and significantly worse survival in this group compared to patients with early disease. In early-stage disease (I, II), the primary treatment offered can be either primary surgical or primary radiotherapy. In contrast, multimodal treatment is often used for patients with advanced stage (III, IV). Notably, all these cases were reviewed by a multidisciplinary team (MDT) comprising the surgical team, radiotherapy team, pathologists, radiologists, and medical oncology team, who were guided by standardized datasets in accordance with the Head and Neck Cancer International Group (HNCIG) consensus recommendations, ensuring consistency in staging, treatment planning, patient demographics [6]. In selected cases, patients were offered both treatment options and made their decision based on personal preferences, particularly considering treatment-related morbidity and the importance of voice preservation.

The majority of patients had glottic laryngeal cancer (40; 63.49%), followed by supraglottic (13; 20.63%), and then subglottic (2; 3.17% ). It is of note that 8 (12.70%) presented with transglottic involvement at diagnosis. This distribution is consistent with global trends, where glottic tumors are the most common, followed by supraglottic and subglottic involvement [2].

Different studies were done to look at the treatment modalities received and the overall survival of patients with laryngeal cancer, especially with the current shift from going with radical surgery to transoral organ preservation surgeries and offering radiotherapy or chemoradiotherapy for early-stage disease [7-11]. According to a retrospective study, longer survival rates were found for patients who underwent surgical treatment for Stages I and IV [9]. For Stages II and III, those who had surgical treatment had longer survival rates, but this was statistically insignificant when compared to those who had similar stages and non-surgical treatment. Another study focusing more on glottic cancer specifically concluded that in early-stage glottic cancer, there was no significant difference in survival rates in patients who underwent surgery vs. radiotherapy alone [12].

In our study, the overall survival rate was higher in the non-surgical group of patients; this does not align with the global literature, where we see that non-surgical and surgical patients have equal overall survival outcomes and, in some instances, even better outcomes, like with Stage IVa disease [9,11,13,14]. This can be attributed to multiple reasons, including small sample size, selection bias, patient preference, or MDT decisions, which take patient co-morbidities and functional status into consideration during decision-making. Stage distribution can also play a role in this instance, as patients with early-stage cancer were treated with CRT while more of the advanced cases underwent surgery, all of which could have amplified these findings.

Contrary to our study, the results of a study conducted to look into the survival of localized laryngeal cancer based on treatment received from the year 1995 to 2009 were different as compared to our study, as it showed that surgical treatment had better survival outcomes compared to non-surgical treatment [15]. Another study had similar findings [16]. These studies highlight that the treatment in the Gulf and Middle East region might need to be tailored to be specific to the local population and not necessarily follow global preferences.

Another study conducted in Saudi Arabia found that patients who underwent surgical intervention had significantly better overall survival compared to those who received non-surgical treatment [17]. However, similar to our study, they faced issues such as sample size, selection bias, and inadequate data documentation. This highlights a broader regional challenge in data consistency and the need for a unified cancer registry system in the Middle East to support high-quality research.

Limitations

This study is subject to expected biases, including missing data and lack of randomization, as it is a retrospective study. The presented sample size may reduce the statistical power to support and link associations, as well as the generalizability of the study to the entire population. Despite these limitations, this study highlights the need for larger, prospective studies to look further into the treatment options available and their outcomes in the given population.

Another limitation of this study is the inability to perform more detailed survival analysis to identify independent prognostic factors. This was due to the relatively small sample size and incomplete documentation of key clinical variables such as comorbidities, performance status, and smoking history. As a result, potential confounding factors could not be fully accounted for, and the observed survival differences should be interpreted with caution.

This study is the first in the UAE addressing laryngeal cancer, and it provides a good start for future studies with a bigger sample size and with more available data that might yield a better representation of the survival outcomes of laryngeal cancer in the UAE. It would be of great value to do more in-depth research on the details of histopathological grading and markers about laryngeal cancer in the region, and correlate it to the aggressiveness of the disease and related survival outcomes. Future research should include more detailed survival analysis to better understand the influence of clinical and treatment-related factors and to help guide improvements in cancer care strategies within the region.

## Conclusions

This retrospective analysis of patients with laryngeal cancer provides insight into patient demographics and relevant risk factors and investigates the primary treatment modality received and the subunit involved. The observed correlation between stage, treatment received, and survival outcomes provided an interesting area for further research.

## References

[1] Amin MB, Edge SB, Greene FL (2017). AJCC Cancer Staging Manual. 8th ed. AJCC Cancer Staging Manual. 8th ed. Springer.

[2] Fu Z, Lv J (2024). A commentary on 'updated disease distributions, risk factors, and trends of laryngeal cancer: a global analysis of cancer registries'. Int J Surg.

[3] Igissin N, Zatonskikh V, Telmanova Z, Tulebaev R, Moore M (2023). Laryngeal cancer: epidemiology, etiology, and prevention: a narrative review. Iran J Public Health.

[4] Liberale C, Soloperto D, Marchioni A, Monzani D, Sacchetto L (2023). Updates on larynx cancer: risk factors and oncogenesis. Int J Mol Sci.

[5] Menach P, Oburra HO, Patel A (2012). Cigarette smoking and alcohol ingestion as risk factors for laryngeal squamous cell carcinoma at Kenyatta National Hospital, Kenya. Clin Med Insights Ear Nose Throat.

[6] Baliga S, Abou-Foul AK, Parente P (2024). Essential data variables for a minimum dataset for head and neck cancer trials and clinical research: HNCIG consensus recommendations and database. Eur J Cancer.

[7] Mannelli G, Lazio MS, Luparello P, Gallo O (2018). Conservative treatment for advanced T3-T4 laryngeal cancer: meta-analysis of key oncological outcomes. Eur Arch Otorhinolaryngol.

[8] Wolf GT, Fisher SG, Hong WK (1991). Induction chemotherapy plus radiation compared with surgery plus radiation in patients with advanced laryngeal cancer. N Engl J Med.

[9] Répássy GD, Molnár A, Maihoub S, Hargas D, Tamás L (2025). Survival analysis of laryngeal squamous cell cancer, considering different treatment modalities and other factors influencing survival - a monocentric retrospective investigation. Eur Arch Otorhinolaryngol.

[10] Leblanc A, Thomas TV, Bouganim N (2023). Chemoradiation for locoregionally advanced laryngeal cancer. Otolaryngol Clin North Am.

[11] Wushouer A, Li W, Zhang M, Lei D, Pan X (2022). Comparison of treatment modalities for selected advanced laryngeal squamous cell carcinoma. Eur Arch Otorhinolaryngol.

[12] Gupta DK, Ravunnikutty M, Singh SK (2024). A comparative study of short-term phonatory outcomes in primary cases of early glottic cancer treated with radiotherapy versus laser surgery. Egypt J Otolaryngol.

[13] Grover S, Swisher-McClure S, Mitra N (2015). Total laryngectomy versus larynx preservation for T4a larynx cancer: patterns of care and survival outcomes. Int J Radiat Oncol Biol Phys.

[14] Patel SA, Qureshi MM, Dyer MA, Jalisi S, Grillone G, Truong MT (2019). Comparing surgical and nonsurgical larynx-preserving treatments with total laryngectomy for locally advanced laryngeal cancer. Cancer.

[15] Misono S, Marmor S, Yueh B, Virnig BA (2014). Treatment and survival in 10,429 patients with localized laryngeal cancer: a population-based analysis. Cancer.

[16] Cîrstea AI, Berteșteanu ȘVG, Scăunașu RV (2023). Management of locally advanced laryngeal cancer-from risk factors to treatment, the experience of a tertiary hospital from Eastern Europe. Int J Environ Res Public Health.

[17] Alamoudi E, Albalawi H, Almutairi A (2023). Survival and disease-free survival in laryngeal cancer and their associated factors in western Saudi Arabia: a retrospective, single center study. J Cancer Res Ther.

